# Novel method for rapid monitoring of OPFRs by LLE and GC–MS as a tool for assessing biodegradation: validation and applicability

**DOI:** 10.1007/s00216-024-05154-7

**Published:** 2024-01-27

**Authors:** Diana Losantos, Oscar Palacios, María Jesús Berge, Montserrat Sarrà, Gloria Caminal, Alba Eustaquio

**Affiliations:** 1 Department of Chemical, Biological and Environmental Engineering, Universitat Autònoma de Barcelona, Escola d’Enginyeria, Campus Bellaterra, 08193 Cerdanyola del Vallès, Spain; 2 Servei d’Anàlisi Química, Universitat Autònoma de Barcelona, Facultat de Ciències, Campus Bellaterra, 08193 Cerdanyola del Vallès, Spain; 3 Institut de Química Avançada de Catalunya (IQAC), Spanish Council for Scientific Research (CSIC), Jordi Girona 18-26, 08034 Barcelona, Spain

**Keywords:** Emerging pollutants analysis, Bioremediation, Sample preparation, Gas chromatography

## Abstract

Organophosphate flame retardants (OPFRs) are high-production volume chemicals widely present in environmental compartments. The presence of water-soluble OPFRs (tri-n-butyl phosphate (TnBP), tris(2-butoxyethyl) phosphate (TBEP), tris(2-chloroethyl) phosphate (TCEP), tris(2-chloroisopropyl) phosphate (TCPP), and triethyl phosphate (TEP)) in water compartments evidences the struggle of conventional wastewater treatment plants (WWTPs) to effectively eliminate these toxic compounds. This study reports for the first time the use of white-rot fungi as a promising alternative for the removal of these OPFRs. To accomplish this, a simple and cost-efficient quantification method for rapid monitoring of these contaminants’ concentrations by GC–MS while accounting for matrix effects was developed. The method proved to be valid and reliable for all the tested parameters. Sample stability was examined under various storage conditions, showing the original samples to be stable after 60 days of freezing, while post-extraction storage techniques were also effective. Finally, a screening of fungal degraders while assessing the influence of the glucose regime on OPFR removal was performed. Longer chain organophosphate flame retardants, TBP and TBEP, could be easily and completely removed by the fungus *Ganoderma lucidum* after only 4 days. This fungus also stood out as the sole organism capable of partially degrading TCEP (35% removal). The other chlorinated compound, TCPP, was more easily degraded and 70% of its main isomer was removed by *T. versicolor*. However, chlorinated compounds were only partially degraded under nutrient-limiting conditions. TEP was either not degraded or poorly degraded, and it is likely that it is a transformation product from another OPFR’s degradation. These results suggest that degradation of chlorinated compounds is dependent on the concentration of the main carbon source and that more polar OPFRs are less susceptible to degradation, given that they are less accessible to radical removal by fungi. Overall, the findings of the present study pave the way for further planned research and a potential application for the degradation of these contaminants in real wastewaters.

**Graphical Abstract:**

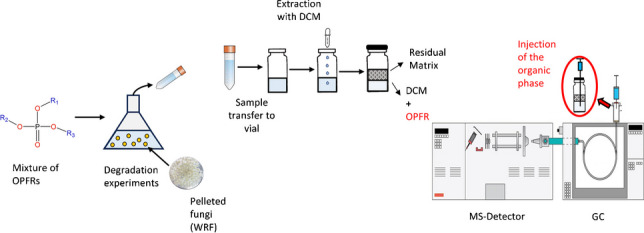

**Supplementary Information:**

The online version contains supplementary material available at 10.1007/s00216-024-05154-7.

## Introduction

Organophosphate flame retardants (OPFRs) encompass a wide range of esters of phosphoric acid characterized by their structural diversity—featuring either alkyl or haloalkyl substituents [1, 2]—and physicochemical properties that make them effective fire-inhibiting agents [3]. As a result, halogenated OPFRs are used as additives in a variety of commercial products such as foams, textiles, plastics, electronics, and furniture, while non-halogenated OPFRs serve as plasticizers and antifoaming agents [4, 5]. They have lately gained popularity as an alternative against polybrominated diphenyl ethers (PBDEs) flame retardants, after the ban on the latter in the European Union in 2009 [6, 7]. Consequently, many OPFRs are now high-production-volume chemicals [8], with an estimated global production of 2,800,000 tons in 2018 and an anticipated annual increase of 15% [9, 10].

As additives, they are not chemically bonded to their base materials and can easily diffuse into surrounding environments during production, use, and end of life stages [3, 9]. The distinct physicochemical properties between each compound determine their distribution between air, water, and sediment compartments [11]. Five OPFRs (tri-n-butyl phosphate (TnBP), tris(2-butoxyethyl) phosphate (TBEP), tris(2-chloroethyl) phosphate (TCEP), tris(2-chloroisopropyl) phosphate (TCPP; usually composed of some isomers, mostly tris(1-chloro-2-propyl) phosphate TCIPP/TCPP-IS1), and triethyl phosphate (TEP)) have been chosen for this study based on their high solubility in water and low volatility (refer to Table S.1); making them more likely to persistently occur in water compartments. These five OPFRs have dangerous traits when exposed to living beings. TEP exhibited potential neurotoxic and mutagenic effects at high doses [12],TBP is neurotoxic and irritating even at low concentrations [13, 14],TBEP is hepatotoxic and carcinogenic [15, 16],and, although TCEP has been currently listed as a priority controlled pollutant in many countries [3] and replaced by TCPP as a safer flame retardant, they are both carcinogenic and neurotoxic [15, 17], and TCEP production has not yet been prohibited [18].

The main identified sources of contamination in water compartments stem from domestic and industrial wastewaters discharges [1, 2, 19]. This situation highlights a challenge: conventional wastewater treatment plants (WWTPs) struggle to effectively eliminate these compounds, which have been consistently identified as prevalent in WWTP effluents [1, 2, 20, 21], especially the chlorinated ones [22–24]. The detection of these OPFRs also in drinking water samples [1, 2, 9, 24, 25] underscores a straight pathway for human exposure.

The use of fungi, specifically white-rot fungi (WRF), offers a promising, cost-effective, and environmentally friendly approach for the removal of OPFRs, as these microorganisms can constitutively degrade a series of contaminants, even at trace concentrations [26]. They achieve this through a co-metabolic pathway with the aid of extracellular ligninolytic enzymes which are non-specific and can act over a wide range of substrates through the generation of radicals [27, 28], and the assistance of redox mediators produced for electron transfer [29]. In many cases, their versatile intracellular system (cytochrome P450) is responsible for the degradation of the contaminants [30]. As these contaminants undergo degradation, the resulting products may become more accessible to other microorganisms, facilitating the cycling of carbon through the biosphere [31].

The purpose of this work was to make an initial evaluation of the ability of different WRF to degrade water-soluble OPFRs. To accomplish this, the development of a robust quantification method that would allow efficient and rapid monitoring of these contaminant’s concentrations while accounting matrix effects was deemed necessary. When reviewing trends in the last year for analyzing these compounds in water samples (refer to Table 1), it is clear that solid-phase extraction (SPE) is the preferred technique for mitigating matrix effects before sample analysis. However, it is worth noting that when performing SPE, the use of internal standards is recommended, which, in the case of OPFRs, can be expensive and impractical when analyzing a substantial number of samples as required when developing wastewater treatment processes.

**Table 1:** Extraction and detection techniques for OPFRs determination in water samples over the year 2023

Identified compounds	Matrix	Extraction technique	Detection	Column	Injection mode	Reference
TnBP, TBEP, TCEP, TCPP (TCIPP)	Drinking water	SPE	GC–MS	DB-5MS (30 m × 0.25 mm × 0.25 µm)	Splitless	[9]
TnBP, TBEP, TCEP, TCPP (TCIPP), TEP	WWTP effluent; rain water	SPE	LC–MS/MS	Eclipse Plus C18 (100 mm × 2.1 mm × 1.8 µm)		[21]
TnBP, TBEP, TCEP, TCPP	Surface water	SPE	GC–MS	DB-5MS (30 m × 0.25 mm × 0.25 µm)	Splitless	[22]
TnBP, TBEP, TCEP, TCPP, TEP	Drinking water; raw water	SPE	LC–MS/MS	Eclipse Plus C18 (100 mm × 2.1 mm × 2.7 µm)	Splitless	[1]
TnBP, TBEP, TCEP, TCPP (TCIPP), TEP	Drinking water; raw water; tap water	SPE	LC–MS	ACQUITY UPLC HSS T3 (100 mm × 2.1 mm × 1.8 µm)		[25]
TnBP, TBEP, TCEP, TCPP (TCIPP), TEP	River water	SPE	LC–MS/MS	Xbridge BEH-C18 XP (100 mm × 4.6 mm × 2.5 µm)		[3]
TnBP, TBEP, TCEP, TCPP	Drinking water; raw water	SPE	GC-QTOF-MS	HP-5MS (30 m × 0.25 m × 0.25 mm)		[32]
TCEP, TCPP	Surface water; underground water; WWTP effluent	SPE	LC–MS	ACQUITY UPLC BEH C18 (50 mm × 2.1 mm × 1.7 µm)		[2]

Thus, the present study aimed to develop and validate a sample preparation and analysis procedure for OPFRs and test it as a tool for assessing biodegradation. A liquid–liquid extraction was performed and the resulting organic-contaminant-rich phase was injected into a GC–MS system. This protocol demonstrated its simplicity, cost-effectiveness, speed, and reliability in quantifying OPFRs under conditions matching the experimental setup. On a side note, stability of the samples before and after extraction, under different storage conditions, was studied to determine the compounds’ stability over time. Finally, a screening was performed to identify fungal degraders, providing an initial insight into which WRF can degrade the tested contaminants, and shedding light on the potential degradation mechanisms. To the best of our knowledge, this is the first time that fungal degradation of a mixture of OPFRs has been reported, and results pave the way for further planned research and a potential application on the degradation of these contaminants in real wastewaters.

## Materials and methods

### Microorganisms and media

*Trametes versicolor* ATCC 42530 was acquired from the American Type Culture Collection, *Ganoderma lucidum* FP-58537-Sp was obtained from the United States Department of Agriculture Collection (Madison, WI), and *Pycnoporus sanguineus* CS43 was gently provided by the Environmental Bioprocesses Group of the Institute of Technology and Higher Studies of Monterrey (México). The strains were maintained by subculturing malt extract agar plates (pH 4.5) at 25 °C every 30 days. Mycelial suspensions and pellets were prepared in malt extract, according to methodology described elsewhere [33].

The defined medium (pH 4.5) used for validation and degradation experiments contained per liter: 8 g glucose, 3.3 g ammonium tartrate, 1.168 g dimethyl succinate, 10 mL micronutrients, and 100 mL macronutrients [34].

### Reagents

OPFRs standards tri-n-butyl phosphate (TnBP ≥ 99%), tris(2-butoxyethyl) phosphate (TBEP 94%), tris(2-chloroethyl) phosphate (TCEP 97%), triethyl phosphate (TEP ≥ 99.8%), and a mixture of isomers (TCPP) containing 66.9% of tris(1-chloro-2-propyl) phosphate (TCIPP/TCPP-IS1), 26.4% of bis(1-chloro-2-propyl)(2-chloropropyl) phosphate (TCPP-IS2), and 4.2% of (1-chloro-2-propyl) bis(2-chloropropyl) phosphate (TCPP-IS3) were acquired from Merck KGaA (Darmstadt, Germany). Methanol (MeOH) GC–MS grade was purchased from Fisher Scientific (NH, USA). Dichloromethane (DCM) GC–MS grade was obtained from Merck KGaA (Darmstadt, Germany). A working standard solution was prepared for analytical purposes by diluting all five standards in methanol up to a concentration of ≈250 mg L^−1^. Also, a stock solution of all five OPFRs at a concentration of ≈10,000 mg L^−1^ for TBP and TBEP and ≈5000 mg L^−1^ for TEP, TCEP, and TCPP in methanol was prepared for fungal degradation experiments. Working standard and stock solutions were stored in the dark at − 20 °C until use. D-(+)-Glucose (C_6_H_12_O_6_) and aluminum potassium sulfate dodecahydrate (AlK(SO_4_)_2_ · 12H_2_O) were purchased from Acros Organics (NJ, USA). Ammonium tartrate dibasic ((NH_4_)_2_C_4_H_4_O_6_), 2,2-dimethyl succinic acid (C_6_H_10_O_4_), nitrilotriacetic acid (C_6_H_9_NO_6_), and magnesium sulfate heptahydrate (MgSO_4_ · 7H_2_O) were obtained from Merck KGaA (Darmstadt, Germany). Manganese (II) sulfate monohydrate (MnSO_4_ · H_2_O), cobalt (II) sulfate heptahydrate (CoSO_4_ · 7H_2_O), zinc sulfate heptahydrate (ZnSO_4_ · 7H_2_O), copper(II) sulfate pentahydrate (CuSO_4_ · 5H_2_O), sodium molybdate dihydrate (Na_2_MoO_4_ · 2H_2_O) and calcium chloride (CaCl_2_) were purchased from ITW Panreac (Barcelona, Spain). Iron (II) sulfate heptahydrate (FeSO_4_ · 7H_2_O), calcium chloride dihydrate (CaCl_2_ · 2H_2_O), boric acid (H_3_BO_3_), sodium chloride (NaCl) and potassium dihydrogen phosphate (KH_2_PO_4_) were acquired from Scharlab (Barcelona, Spain). 

### Analytic method

#### Sample preparation

Filtered samples went through a liquid–liquid extraction process to eliminate the interference due to salts contained in the medium. Extraction was performed in crimp neck vials. Dichloromethane DCM (0.5:1 v/v regarding sample volume) was added as an extraction solvent. Methanol was also added (0.05:1 v/v regarding sample volume) to equalize the amount of this between analyzed samples and standard solutions (refer to Table S.2). Vials were then encapsulated, vortex-mixed for 1 min, and centrifugated at 4100 rpm for 20 min, to separate the organic phase (containing the contaminant) from the medium. Such phase was injected from the vial to the GC–MS by programming the needle height for this purpose.

#### Component’s identification and quantification

Identification and quantification were performed by means of an Agilent HP 6890 Series II gas chromatograph coupled to an HP5973 electron ionization mass spectrometric detector (GC/MS; Agilent Technologies, CA, USA). The system was equipped with automated injection and sampling (Combi PAL®, CTC Analytics, Zwingen, Switzerland). A ZB-5 chromatographic column (30 m × 0.25 mm i.d × 0.25 µm film thickness; Phenomenex, CA, USA) was used for components separation. The inlet temperature was maintained at 310 °C, while the MS ion source and quadrupole temperatures were set at 230 °C and 150 °C, respectively. Helium served as the carrier gas at a constant flow rate of 0.9 mL·min^−1^. The oven’s temperature program was set from 40 °C (1-min hold) to 310 °C (5-min hold) at 20 °C·min^−1^. A Pulsed Splitless (20 psi for 1 min) injection mode was chosen to enhance the method’s sensitivity. An Agilent 5183–8711 inlet liner of glass wool (single taper, split injection type) was used in the injector port. The injection volume was 1.0 µL.

An initial components identification was carried out by registering the working standard solution in Full Scan mode (m/z = 30–300). Mass spectra of each analyte were obtained. Then, compounds were identified by comparison of each spectrum with Wiley’s 7n Registry of Mass Spectra [35]. Two parameters were chosen as identification criteria: Quantifying (q) and qualifying (c) ions, selected as the two most intense and characteristic ions for each compound; and the retention time. For quantification purposes, the acquisition mode was switched to Single Ion Monitoring (SIM) mode, to enhance the sensitivity of the method, where quantifying and qualifying ions’ masses were fixed. Matrix-matched calibration curves allowed to set two compliance parameters to be met by all the measured samples:

- The ratio between the area of the quantifying (Aq) and the qualifying ion (Ac), where a tolerance was set for each compound from the mean of *n* = 5 matrix-matched calibration curves.
- The retention time, for which a restrictive tolerance was set. Only values within three times the relative standard deviation (RSD) from the mean of *n* = 5 matrix-matched calibration curve retention times were considered acceptable.

#### Method validation

Validation was performed for the entire method, including sample preparation. Thus, all the samples used for validation underwent the same extraction process. Also, quantification was performed with matrix-matched calibration curves.

Limits, linearity, and stability of the sequence were evaluated by means of standard solutions prepared by spiking defined medium with aliquots of the working standard solution. The obtained concentrations for each standard solution are dependent on the standard’s starting purity and may vary a few from compound to compound. Also, in the case of TCPP, concentrations of each isomer will vary according to their mass fraction in the mix (refer to Table S.2). Standard solutions then underwent the same extraction process as stated above.

The limit of detection (LOD) was determined experimentally for each compound, by preparing standard solutions at concentrations of ~ 0.5, 0.25, 0.125, 0.05, 0.025, 0.012, and 0.006 mg·L^−1^. The LOD was defined as the lowest concentration at which 3 parameters were met: (a) the compound’s retention time ± a tolerance of 0.5%, (b) the ratio between the areas of the quantifying and the qualifying ion ± a tolerance of 15%, and (c) a signal-to-noise ratio (*S*/*N*) above 3.

The limit of quantification (LOQ) was assigned to the lowest concentration of the calibration curve (1.25 mg·L^−1^) as it complied with the research purposes. Acceptance criteria for the LOQ were set in terms of the relative standard deviation (RSD) < 15% between five different matrix-matched calibrations; recovery, defined as the ratio between the average of n values and the theoretical value, amid 80 and 120% [36]; and a *S*/*N* above 10.

Linearity was evaluated daily by matrix-matched calibration curves of 6 points (~ 1.25, 2.5, 5, 7.5, 10, and 12.5 mg·L^−1^, chosen as they cover the range of expected concentrations in the fungal degradation experiments) throughout the whole validation period. Acceptability criteria for linearity were met by the coefficient of determination *R*^2^ > 0.99 and residual error of estimation within the target <  ± 15% and <  ± 20 for the LOQ [36].

The stability of the sequence was measured as a response drift throughout the batch, by placing standard solutions spiked at concentrations of ~ 2.5, 5, 7.5, and 10 mg·L^−1^ of each compound at the end of each sequence and a 5 mg·L^−1^ standard solution in the middle of the sequence. The sequence was considered stable when the recovery was between 85 and 115% at each tested concentration [36].

Precision was expressed in terms of repeatability and intra-laboratory reproducibility. Repeatability was expressed as the RSD of 3 consecutive measurements of the same sample. In this case, samples used were obtained after inoculating pellets of the three tested fungi in defined medium spiked with a mixture of the OPFRs at a concentration of 5 mg·L^−1^. Samples were taken after 6 days of inoculation and filtered through syringe-driven filters of hydrophilic PTFE 0.2 µm (Millipore Millex-LG, Merck KGaA) for biomass removal. Intra-laboratory reproducibility was also determined in terms of the RSD, but in this case from standard solutions (at a concentration of ~ 5 mg·L^−1^ for each OPFR) of *n* = 5 different matrix-matched calibration curves. The acceptance criterion was set at an RSD < 15% for both parameters.

Accuracy was evaluated by spiking samples used for measuring repeatability, with the working standard solution at a concentration of ~ 2.5 mg·L^−1^, and calculated as indicated in Eq. 1:

1 $$Accuracy\left(\%\right)=\frac{\overline{\left[MC\right]}+\left[SC\right]}{\stackrel{-}{\left[FC\right]}}*100$$

where $\overline{\left[MC\right]}$ is the measured concentration before spiking (average of *n* = 3 samples); $[SC]$ is the spiked concentration; and $\overline{\left[FC\right]}$ is the fortified concentration (average of *n* = 3 samples).

If the accuracy was between 85 and 115% [36], the method was valid for this parameter.

##### Blank quality control

A blank quality control to test the specificity of the method was performed by analyzing samples containing only defined medium, after performing the extraction process. Samples were analyzed in triplicate.

##### Carryover study

The carryover effect over the instrument was studied by analyzing blank dichloromethane samples after the most concentrated sample of the calibration curve.

### Sample stability tests

Assessing the stability of the samples is relevant because, although it would be optimal to measure the samples immediately after taking them, there are external factors that can sometimes prevent this from happening and it is important to know the working window within which samples are still viable, especially when long-term studies are planned.

First, stability was tested for samples obtained after 14 days of fungal degradation (refer to the next section), which were filtered and stored in the freezer for 60 days. These tests were performed with the three tested fungal matrices. On the other hand, the stability of the samples after extraction with DCM was also tested. This was done by measuring the defined medium spiked with the 5 contaminants at the same initial concentrations as fungal degradation experiments (TBP and TBEP = 10 mg·L^−1^; TEP, TCEP, and TCPP = 5 mg·L^−1^), at different times and temperature conditions. Three conditions were chosen: (a) injection after 1 day in the GC–MS sample tray (reinjection stability); (b) injection after 1 week of storage at the refrigerator (short-term stability); and (c) injection after the sample was frozen for 3 weeks and then measured right after thawing (long-term stability). These conditions were chosen as they were likely to be encountered during sample storage and analysis in the present work.

All stability tests were performed by duplicate, and stability was evaluated by means of a degradation percentage, calculated as follows:

2 $$Degradation\;\left(\%\right)=\left|\frac{{\overline X}_{t=0}-{\overline X}_{t=F}}{{\overline X}_{t=0}}\right|\;\ast\;100$$

where ${\overline X}_{t=0}$ is the average concentration (*n* = 2) at time 0 and ${\overline X}_{t=F}$ is the average concentration (*n* = 2) after the stability interval.

Samples were defined as stable when the degradation was below 15%.

### Screening of fungal degraders

Results obtained when testing repeatability and accuracy of the samples after fungal contact gave us a first glance of the degradation process, where it became evident that TBP and TBEP were easily degraded, which was not the case for TEP, TCEP, and TCPP. Thus, it was decided (a) to reduce the concentrations of the not so degradable compounds and (b) to further study the effect that glucose addition might have on degradation.

Degradation experiments were performed in 500-mL Erlenmeyer flasks containing 100 mL of sterile defined medium, fortified with the stock solution up until a concentration of 10 mg·L^−1^ for TBP and TBEP and 5 mg·L^−1^ for TEP, TCEP, and TCPP. The experiments were conducted using either *T. versicolor*, *G. lucidum*, or *P. sanguineus* as inoculum. Fungal pellets of each microorganism were transferred into the flasks, at a concentration equivalent to ~ 3.5 g DCW·L^−1^ (dry cell weight). The cultures were incubated at 25 °C under continuous orbital shaking (135 rpm) for 14 days. Each set of trials was run in triplicate.

To test the influence of the glucose regime, samples were taken at the start and after 4 days of experiment to evaluate immediate degradation under optimal glucose conditions. At day 4, glucose was supplemented again at a concentration of 3 g/L. After 14 days of experiment, the fungi were under nutrient-limiting conditions, and samples were taken again. Two milliliters was withdrawn at the defined sampling times for the analysis of the residual concentrations of the OPFRs. Samples were then filtered through syringe-driven filters of hydrophilic PTFE 0.2 µm (Millipore Millex-LG, Merck KGaA) for biomass removal, and frozen until its use.

## Results and discussion

### Analytic method

#### Component’s identification and quantification

The method allowed the correct identification of all five OPFRs, for which mass spectra were obtained (refer to Figure S.1). The identification and quantification criteria set for this study are depicted in Table 2.

**Table 2:** Identification and quantification criteria for testing the OPFRs in GC–MS. Values obtained for the retention time and the ratio between the areas of the quantifying and the qualifying ion were obtained from *n* = 5 matrix-matched calibration curves

Compound	Quantifying ion (m/z)	Qualifying ion (m/z)	Retention time (min) ± 3RSD (%)^a^	Aq/Ac ratio ± tolerance (%)
TEP	99.0	155.0	6.67 ± 0.18%	1.08 ± 5%
TBP	98.9	155.0	10.24 ± 0.07%	4.67 ± 5%
TCEP	248.9	251.0	10.94 ± 0.08%	1.56 ± 5%
TCPP-IS1	98.9	125.0	11.16 ± 0.05%	0.89 ± 10%
TCPP-IS2	98.9	125.0	11.23 ± 0.05%	1.49 ± 10%
TCPP-IS3	98.9	125.0	11.28 ± 0.06%	3.68 ± 10%
TBEP	57.0	85.0	13.94 ± 0.05%	2.14 ± 15%

*RSD*, relative standard deviation (%); *Aq*, quantifying ion area; *Ac*, qualifying ion area

^a^Criteria taken from Domènech et al. [37]

**Fig. 1:**
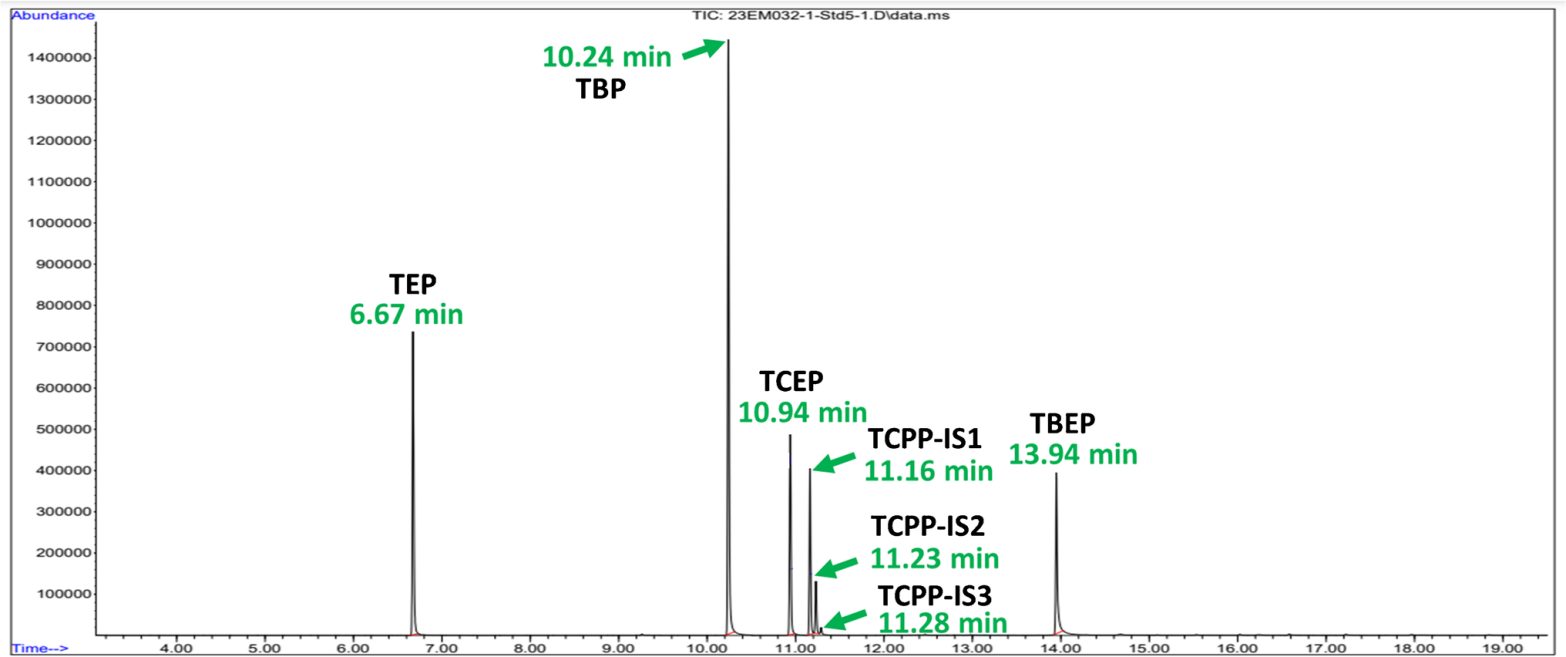
Chromatogram of a standard solution spiked at 5 mg·L^*−*^^1^

Figure 1 depicts a chromatogram in SIM mode of a calibration standard solution, spiked at a concentration of 5 mg·L^−1^ for each OPFR and purified as explained before. The total run time was of 19.50 min, with a solvent delay period of 3 min. All seven peaks were easily detected without any overlap between them.

#### Method validation

##### Limits, linearity, and stability of the sequence

Concentrations determined as the LOD of each compound (refer to Table 3) satisfy the three parameters established in the “Materials and methods” section, with TBP, TCPP-IS2, and TCPP-IS3 being the most sensitively detected compounds (LOD of 0.012 mg·L^−1^). The LOQ could be measured with a correct intra-day reproducibility (RSD < 10% for each compound) while recovery values of the analyte were also appropriate (93–119%). A signal-to-noise ratio above 10 was also obtained in each case (data not displayed).

**Table 3:** Limits of detection (LOD) and quantification (LOQ)

Compound	LOD (mg·L^−1^)	LOQ (mg·L^−1^)	RSD of the LLOQ (%) *n* = 5	Recovery of the analyte at the LOQ (%) *n* = 5
TEP	0.05	1.29	6.36	100.62
TBP	0.012	1.18	8.95	93.39
TCEP	0.025	1.25	9.86	98.56
TCPP-IS1	0.025	0.83	8.13	95.16
TCPP-IS2	0.012	0.33	7.05	101.94
TCPP-IS3	0.012	0.05	8.79	112.42
TBEP	0.25	1.27	7.74	118.90

Results in Table 4 prove that the method is linear between the range of ~ 1.25–12.5 mg·L^−1^. Data obtained from five different matrix-matched calibrations depict an average of *R*^2^ > 0.99 in all cases. The ranges obtained for the calculated residual errors of estimation were below the set limit of 15% at every concentration, and 20% at the LOQ. The sequence is also stable within the measured concentration range, as the average recovery of each analyte was between 100 and 110%.

**Table 4:** Linearity, residual errors of estimation, and sequence stability parameters obtained for *n* = 5 matrix-matched calibrations

Compound	Lineal range (mg·L^−1^)	*s*^2^ ± RSD	Residual error of estimation (%)	Analyte’s recovery (sequence stability) ± RSD (%)
TEP	1.29–12.87	0.997 ± 0.162%	0.16–8.61	106.15 ± 6.49%
			0.9–10.24 (LOQ)	
TBP	1.18–11.83	0.996 ± 0.127%	0.23–11.25	110.05 ± 7.43%
			1.06–15.85 (LOQ)	
TCEP	1.25–12.51	0.996 ± 0.137%	0.12–10.64	108.25 ± 5.77%
			0.87–11.64 (LOQ)	
TCPP-IS1	0.83–8.26	0.997 ± 0.002%	0.09–9.85	107.10 ± 6.29%
			0.28–13.87 (LOQ)	
TCPP-IS2	0.33–3.26	0.997 ± 0.155%	0.14–9.05	107.25 ± 5.36%
			3.99–11.42 (LOQ)	
TCPP-IS3	0.05–0.52	0.999 ± 0.085%	0.06–11.38	109.05 ± 5.74%
			3.10–18.25 (LOQ)	
TBEP	1.27–12.67	0.997 ± 0.088%	0.27–14.55	105.70 ± 9.98
			6.29–17.95 (LOQ)	

##### Precision and accuracy

According to data in Table 5, the method is both accurate and repeatable under the three fungal matrices tested in this work, with an accuracy between 95 and 115% and a repeatability RSD < 10% in all cases. This shows that the extraction method used for sample purification efficiently removes matrix effects at the studied conditions. Intra-laboratory reproducibility was also proved, evidencing the method’s precision.

**Table 5:** Method’s accuracy and precision data

Compound	Accuracy *n* = 3 (%)	Precision	
		Repeatability *n* = 3 (%)	Reproducibility *n* = 5 (%)
*Ganoderma lucidum*			Matrix-matched standard solution (~ 5 mg·L^−1^)
TEP	96.95	0.87	1.72
TBP	102.08	< LOD*	2.36
TCEP	98.88	4.29	2.54
TCPP-IS1	99.08	3.30	2.59
TCPP-IS2	100.74	1.92	2.58
TCPP-IS3	105.13	1.10	4.17
TBEP	112.15	0.26	3.16
*Trametes versicolor*			
TEP	97.68	1.57	
TBP	105.92	4.01	
TCEP	97.62	7.50	
(TCPP)			
TCPP-IS1	97.75	5.49	
TCPP-IS2	98.38	5.80	
TCPP-IS3	97.81	5.03	
TBEP	97.88	5.11	
*Pycnoporus sanguineus*			
TEP	100.53	0.66	
TBP	105.87	0.23	
TCEP	101.46	0.60	
(TCPP)			
TCPP-IS1	101.85	0.48	
TCPP-IS2	102.63	1.38	
TCPP-IS3	104.76	1.75	
TBEP	103.90	0.75	

*Below the limit of detection as TBP was completely degraded after fungal contact

##### Blank quality control

Chromatograms of blank medium samples did not show any interference that might lead to false positive results. Therefore, the method is specific enough.

##### Carryover study

No carryover effects were observed in DCM samples for any of the OPFRs analyzed in this study, which means that the system is clean after each injection, and it is possible to analyze samples sequentially.

### Sample stability tests

Results of Table 6 showed that the tested OPFRs are stable after 60 days of storage, in all the fungal matrices, as degradation is within the set limits. This reinforces the idea that filtering samples and then freezing them are enough to stop fungal activity. In the case of TBP and TBEP, stability could not be predicted as TBP is completely degraded in two out of three of the fungal matrices and TBEP in all of them. However, this gives us an indication that, in case the contaminant is sorbed in the fungal biomass, there is no biomass present that desorbs the contaminant in the liquid sample. On the other hand, it can be deduced that TBP will have the same behavior as in the matrix of *P. sanguineus*, given the similar results between other OPFRs in the three fungal matrices. In the case of TBEP, as this compound has a higher solubility in water, it is most likely that it is stable in the aqueous sample. On a different note, it was noticed that a precipitate appears when freezing the samples, which could affect the extraction efficiency of the process. This precipitation is reversible by tempering and vortex mixing of the samples.

**Table 6:** Stability data obtained for fungal degradation samples after filtering and 60 days of storage in the freezer

Compound	*Ganoderma lucidum*			*Trametes versicolor*			*Pycnoporus sanguineus*		
	${\overline X}_{\mathrm t=0}$ ± SD	${\overline X}_{\mathrm t=\mathrm F}$ ± SD	Degradation (%)	${\overline X}_{\mathrm t=0}$ ± SD	${\overline X}_{\mathrm t=\mathrm F}$ ± SD	Degradation (%)	${\overline X}_{\mathrm t=0}$ ± SD	${\overline X}_{\mathrm t=\mathrm F}$ ± SD	Degradation (%)
TEP	5.52 ± 0.28	5.75 ± 0.34	4.07	6.03 ± 0.23	6.03 ± 0.13	0.03	6.07 ± 0.32	6.08 ± 0.25	0.16
TBP	< LOD	< LOD	n.d	< LOD	< LOD	n.d	2.68 ± 0.15	2.76 ± 0.32	3.17
TCEP	5.10 ± 0.11	4.96 ± 0.10	2.75	6.08 ± 0.11	5.83 ± 0.13	4.19	6.71 ± 0.25	6.22 ± 0.25	7.23
TCPP-IS1	2.61 ± 0.01	2.95 ± 0.01	13.00	1.30 ± 0.10	1.45 ± 0.15	11.92	4.30 ± 0.31	4.42 ± 0.26	2.91
TCPP-IS2	1.94 ± 0.07	2.09 ± 0.06	7.47	0.45 ± 0.01	0.47 ± 0.01	6.74	3.89 ± 0.25	3.74 ± 0.35	3.73
TCPP-IS3	1.44 ± 0.09	1.49 ± 0.16	3.83	< LOD	< LOD	n.d	4.06 ± 0.31	3.58 ± 0.30	11.82
TBEP	< LOD	< LOD	n.d	< LOD	< LOD	n.d	< LOD	< LOD	n.d

${\overline X}_{\mathrm t=0}$, average concentration (*n* = 2) at time 0; ${\overline X}_{\mathrm t=\mathrm F}$, average concentration (*n* = 2) after the stability interval; SD, standard deviation (*n* = 2)

In the case of the results of Table 7, extracted samples maintained good stability for all OPFRs after reinjection and in the short term, with optimal degradation percentages. Although degradation of extracted samples after freeze/thaw processes is within the set acceptance criterion, it seems that three weeks is the limit time for freezing extracted samples and viability cannot be ensured after this time.

**Table 7:** Stability data obtained under different storage conditions with samples spiked at 10 mg·L^−1^ for TBP and TBEP, and 5 mg·L^−1^ for TEP, TCEP, and TCPP

Compound	Re-injection (1 day)			Short-term (refrigerator for 1 week)			Freeze/thaw (freezer for 3 weeks)		
	${\overline X}_{\mathrm t=0}$ ± SD	${\overline X}_{\mathrm t=\mathrm F}$ ± SD	Degradation (%)	${\overline X}_{\mathrm t=0}$ ± SD	${\overline X}_{\mathrm t=\mathrm F}$ ± SD	Degradation (%)	${\overline X}_{\mathrm t=0}$ ± SD	${\overline X}_{\mathrm t=\mathrm F}$ ± SD	Degradation (%)
TEP	5.64 ± 0.07	5.41 ± 0.21	4.08	5.64 ± 0.07	5.95 ± 0.06	5.41	4.68 ± 0.17	5.33 ± 0.37	14.06
TBP	9.24 ± 0.01	8.83 ± 0.30	4.44	9.24 ± 0.01	8.78 ± 0.09	5.03	9.14 ± 0.03	10.32 ± 0.30	12.97
TCEP	6.15 ± 0.01	6.04 ± 0.03	1.79	6.15 ± 0.01	6.31 ± 0.03	2.69	5.83 ± 0.20	6.63 ± 0.23	13.67
TCPP-IS1	4.68 ± 0.22	4.28 ± 0.27	8.45	4.68 ± 0.22	4.76 ± 0.15	1.82	5.07 ± 0.01	5.42 ± 0.28	6.91
TCPP-IS2	4.52 ± 0.21	4.19 ± 0.24	7.30	4.52 ± 0.21	4.64 ± 0.15	2.65	4.86 ± 0.03	5.43 ± 0.28	11.77
TCPP-IS3	4.30 ± 0.19	4.04 ± 0.15	5.94	4.30 ± 0.19	4.58 ± 0.09	6.52	4.73 ± 0.01	5.23 ± 0.32	10.63
TBEP	10.14 ± 0.11	9.91 ± 0.25	2.32	10.14 ± 0.11	10.19 ± 0.25	0.44	9.59 ± 0.17	10.84 ± 0.20	12.94

${\overline X}_{\mathrm t=0}$, average concentration (*n* = 2) at time 0; ${\overline X}_{\mathrm t=\mathrm F}$, average concentration (*n* = 2) after the stability interval; SD, standard deviation (*n* = 2)

### Screening of fungal degraders

**Fig. 2:**
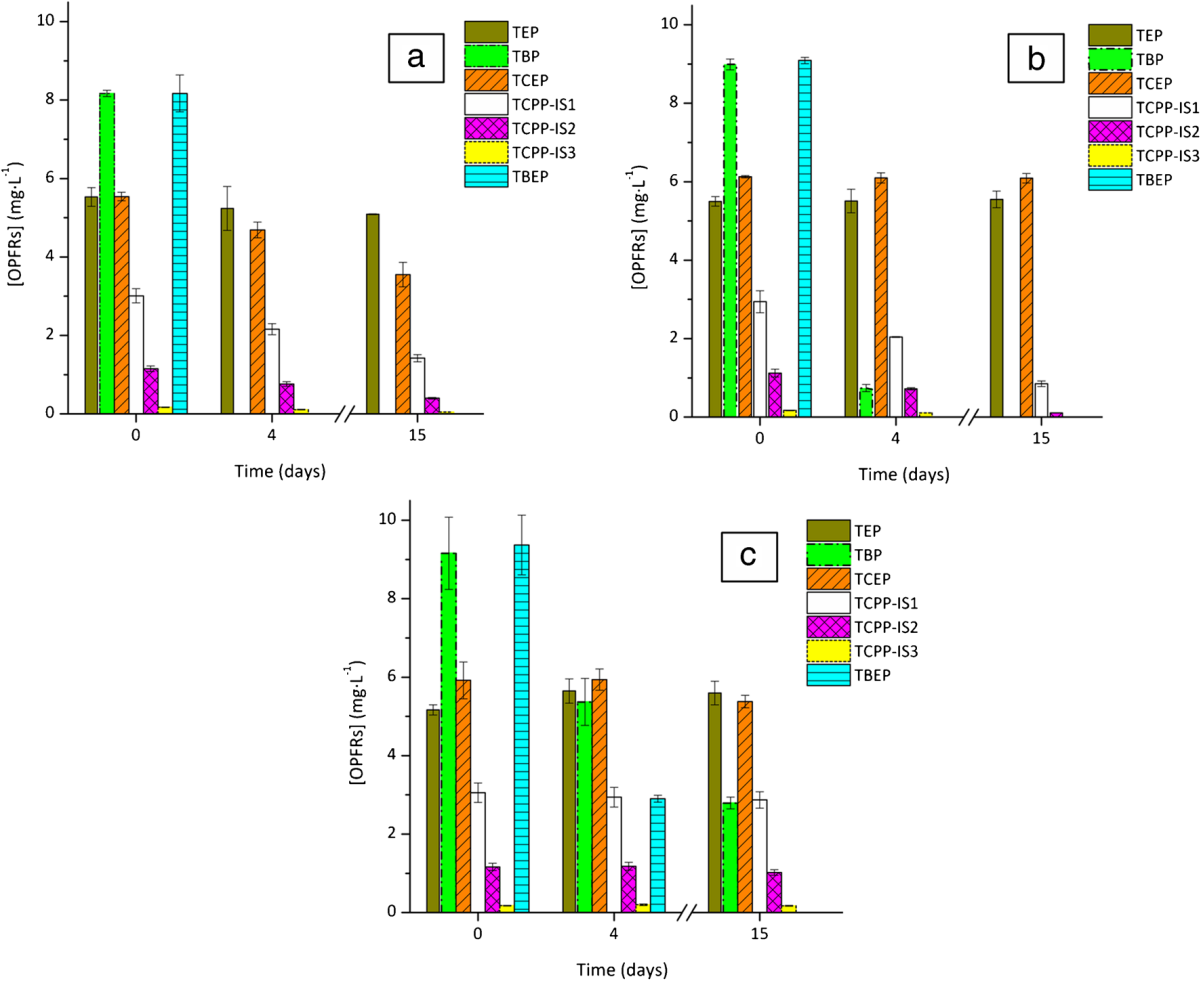
OPFRs degradation profile by the fungi: **a** *G. lucidum*; **b** *T. versicolor*; and **c** *P. sanguineus* after 4 and 15 days of experiment

Results shown in Fig. 2 are very significant as not only they allow to evaluate the performance of three different fungal candidates for degrading OPFRs, while assessing the influence the glucose regime has on this performance, but also evidenced that the method works as an efficient tool to follow the degradation profile of all contaminants, as RSDs between the triplicates were very low in each case, and the ratio between the quantifying and qualifying ions was always checked.

These first results suggest that ligninolytic fungi have the intrinsic ability to degrade some OPFRs that are not so easily degraded by conventional treatments. By looking further into the individual performance of each fungus, *Ganoderma lucidum* exhibited superior capabilities in terms of TBP and TBEP degradation, although *Trametes versicolor* was also able to efficiently remove these compounds in a short time. This ability has been supported by other studies that highlight these fungi proficiency in breaking down xenobiotic compounds [38–44]. *Pycnoporus sanguineus*, in contrast, is the least effective degrader of these contaminants. Given that the main characteristic of this strain is to have a very high and stable laccase enzymatic activity [45], it is likely that this enzyme is not the main precursor for OPFRs degradation.

*Ganoderma lucidum* also stands out as the sole organism capable of degrading TCEP. However, this degradation is only partial (a maximum removal of 35% was obtained) and occurs mostly only under nutrient-limiting conditions. This would indicate that the degradation of chlorinated OPFRs is conditioned by the concentration of the carbon source in the medium. Interestingly, the other chlorinated OPFR, TCPP, is more susceptible to degradation. In this case, *Trametes versicolor* proved to be the most efficient fungus for degrading this pollutant (a removal of 70% was obtained). Nevertheless, the complete degradation of this compound also remains unachieved, thus highlighting the low biodegradability of chlorinated compounds. When looking at the isomers of TCPP, isomer 3 vanishes entirely, unlike isomers 1 and 2. This is due to the initial concentration of isomer 3 being quite low. Nonetheless, when considering the rate of elimination, isomer 1 is actually eliminated more rapidly than isomer 2, and isomer 2 degradation rate surpasses the one of isomer 3. This can be explained as degradation at these concentration levels follows pseudo-first-order kinetics.

In the case of TEP, only 8% was removed by *G. lucidum*, while in the other scenarios, its concentration tended to increase, raising the possibility for it to be a transformation product from the other OPFRs degradation, with a very low biodegradability. This should be verified in the future.

According to the results, the polarity of these OPFRs plays a key role for their fungal degradation, since it was observed that the compounds with the highest polarity are the most difficult to degrade, while the least polar ones are more susceptible to fungal treatments (refer to table S.1). This behavior has also been observed in other biological treatments [46]. We theorize that this is because fungi degrade these contaminants by taking radicals of them [47], so longer chains will be more available to the fungus and therefore more readily removed. Consequently, TEP and TCEP would be more difficult to degrade.

## Conclusions

The method developed in this study proved to be valid, exact, and reproducible for the analysis of OPFRs under fungal-maintenance matrices, in a range of 1.25–12.5 mg·L^−1^ of each contaminant. The stability of the samples after 60 days of freezing was verified, which reinforces the idea that filtering samples and then freezing them are enough to stop fungal activity. Storage techniques for already extracted samples also proved to be efficient. Thus, stability can be maintained by measuring extracted samples after short-term storage in the refrigerator. While frozen extracted samples can remain viable in the long term, this viability cannot be guaranteed beyond 3 weeks. As for the fungal screening, *Ganoderma lucidum* and *Trametes versicolor* emerge as the best degraders for TBP and TBEP, while *T. versicolor* showed a better performance when degrading TCPP. However, *G. lucidum* emerges as the only organism capable of partially degrading TCEP. These findings suggest two “rules of thumb”: (a) The recalcitrance of chlorinated compounds evidences a degradation process dependent on the concentration of the main carbon source and (b) more polar OPFRs are less susceptible to degradation, given that they are less accessible to radical’s remotion by fungi.

### Supplementary Information

Below is the link to the electronic supplementary material.

Supplementary file1 (DOCX 575 KB)
