# Supplementary material for: Novel method for rapid monitoring of OPFRs by LLE and GC–MS as a tool for assessing biodegradation: validation and applicability
Source: Anal Bioanal Chem. 2024 Jan 27;416(6):1493–504. doi: 10.1007/s00216-024-05154-7 (PMC10861394; doi:10.1007/s00216-024-05154-7)
Supplement: Supplementary file 1 — Supplementary file1 (DOCX 575 KB) [file 216_2024_5154_MOESM1_ESM.docx]

**Supporting information**

Novel method for rapid monitoring of OPFRs by LLE and GC-MS as a tool for assessing fungal degradation: validation and applicability.

Diana Losantos^a^, Oscar Palacios^b^, María Jesús Berge^b^, Montserrat Sarra^a,*^, Glòria Caminal^c^, Alba Eustaquio^b^

^a^ *Department of Chemical, Biological and Environmental Engineering, Universitat Autònoma de Barcelona, Escola d’Enginyeria, Campus Bellaterra 08193 Cerdanyola del Vallès, Spain*

^b^ *Servei d'Anàlisi Química, Universitat Autònoma de Barcelona, Facultat de Ciències, Campus Bellaterra 08193 Cerdanyola del Vallès, Spain*

^c^ *Institut de Química Avançada de Catalunya (IQAC), Spanish Council for Scientific Research (CSIC). Jordi Girona 18-26, 08034 Barcelona, Spain.*

^*^ Corresponding author email: [montserrat.sarra@uab.cat](mailto:montserrat.sarra@uab.cat)

D. Losantos ORCID: 0000-0002-7782-3537

M. Sarra ORCID: 0000-0002-3447-6328

G. Caminal ORCID: 0000-0001-9646-6099

**Table S. 1.** Physicochemical properties of OPFRs analyzed in this work.

| **Compound** | **Abbreviation** | **Water solubility (mg·L^-1^) at 25°C** | **Vapor pressure (mmHg) at 25°C** | **Henry’s Law constant (atm-m^3^ mole^-1^) at 25°C** | **log K_ow_** |
| --- | --- | --- | --- | --- | --- |
| Tributyl phosphate | TBP | 2.80 x 10^2^ | 1.13 x 10^-3^ | 1.50 x 10^-7^ | 4.00 |
| Tris(2-butoxy ethyl) phosphate | TBEP | 1.20 x 10^3^ | 2.10 x 10^-7^ | 1.20 x 10^-11^ | 3.65 |
| Tris(2-chloroethyl) phosphate | TCEP | 7.00 x 10^3^ | 1.10 x 10^-4^ | 3.30 x 10^-6^ | 1.44 |
| Tris(2-chloroisopropyl) phosphate | TCPP | 1.60 x 10^3^ | 1.90 x 10^-6^ | 6.00 x 10^-8^ | 2.59 |
| Triethyl phosphate | TEP | 5.00 x 10^5^ | 2.90 x 10^-1^ | 3.50 x 10^-6^ | 0.80 |

(*ChemSpider*, 2011; Lee et al., 2016; Van der Veen & de Boer, 2012)

**Table S. 2:** Standard solutions and sample preparation.

|  | Standard solutions preparation | | | | | | | Sample preparation | |
| --- | --- | --- | --- | --- | --- | --- | --- | --- | --- |
|  | Std-12.5 | Std-10 | Std-7.5 | Std-5 | Std-2.5 | Std-1.25 | Order of addition | Sample | Order of addition |
| Aliquot from stock solution (µL) | 50 | 40 | 30 | 20 | 10 | 5 | 3 |  |  |
| Aliquot of methanol (µL) | 0 | 10 | 20 | 30 | 40 | 45 | 2 | 50 | 2 |
| Medium/sample volume (µL) | 1000 | 1000 | 1000 | 1000 | 1000 | 1000 | 1 | 1000 | 1 |
| DCM volume (µL) | 500 | 500 | 500 | 500 | 500 | 500 | 4 | 500 | 3 |
| TEP concentration (mg·L^-1^) | 12.87 | 10.30 | 7.72 | 5.15 | 2.57 | 1.29 |  |  |  |
| TBP concentration (mg·L^-1^) | 11.83 | 9.46 | 7.10 | 4.73 | 2.37 | 1.18 |  |  |  |
| TCEP concentration (mg·L^-1^) | 12.51 | 10.01 | 7.51 | 5.01 | 2.50 | 1.25 |  |  |  |
| TCPP concentration (mg·L^-1^) | 12.35 | 9.88 | 7.41 | 4.94 | 2.47 | 1.24 |  |  |  |
| TCPP-IS1 concentration (mg·L^-1^) | 8.26 | 6.61 | 4.96 | 3.30 | 1.65 | 0.83 |  |  |  |
| TCPP-IS2 concentration (mg·L^-1^) | 3.26 | 2.61 | 1.96 | 1.30 | 0.65 | 0.33 |  |  |  |
| TCPP-IS3 concentration (mg·L^-1^) | 0.52 | 0.41 | 0.31 | 0.21 | 0.10 | 0.05 |  |  |  |
| TBEP concentration (mg·L^-1^) | 12.67 | 10.13 | 7.60 | 5.07 | 2.53 | 1.27 |  |  |  |


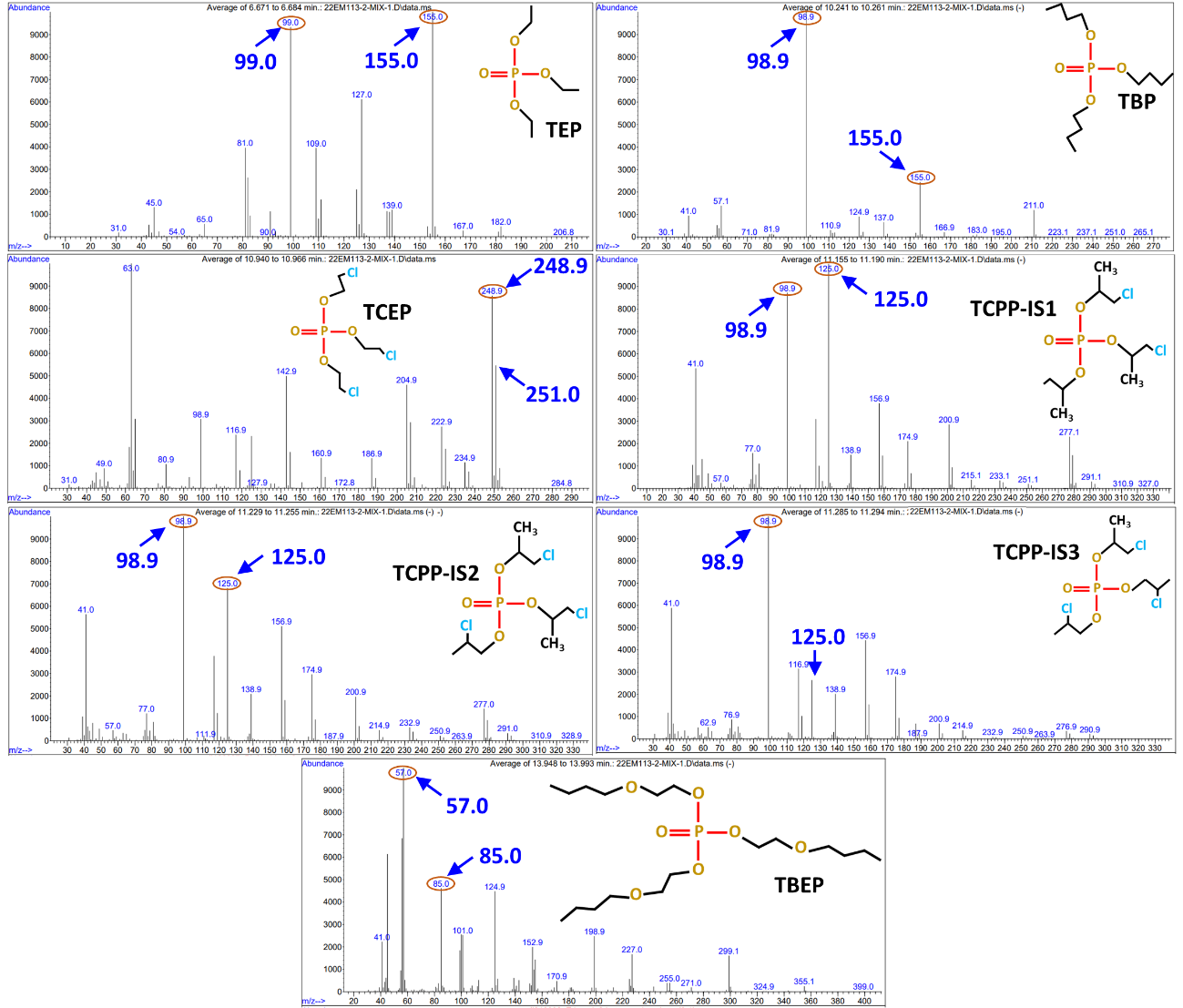


**Figure S. 1:** GC-MS spectrum of each of the identified OPFRs. Each spectrum was obtained by registering a working standard solution of ≈250 mg·L^-1^ under Full Scan mode.

**References**

*ChemSpider*. (2011). http://www.chemspider.com/

Lee, S., Jeong, W., Kannan, K., & Moon, H. B. (2016). Occurrence and exposure assessment of organophosphate flame retardants (OPFRs) through the consumption of drinking water in Korea. *Water Research*, *103*, 182–188. https://doi.org/10.1016/j.watres.2016.07.034

Van der Veen, I., & de Boer, J. (2012). Phosphorus flame retardants: Properties, production, environmental occurrence, toxicity and analysis. *Chemosphere*, *88*(10), 1119–1153. https://doi.org/10.1016/j.chemosphere.2012.03.067
